# Strategies to reduce attrition in randomised trials

**DOI:** 10.1186/1745-6215-12-S1-A128

**Published:** 2011-12-13

**Authors:** Valerie Brueton, Jayne Tierney, Sally Stenning, Irwin Nazareth, Sarah Meredith, Seeromanie Harding, Greta Rait

**Affiliations:** 1 MRC General Practice Research Framework, London, WC2B 6NH, UK; 2 UCL Research department Primary Care and Population Health, London, NW3 2FP, UK; 3 MRC Clinical Trials Unit, London, WC2B 6NH, UK; 4 MRC Social & Public Health Sciences Unit, Glasgow, G12 8RZ, UK

## Background

Attrition from randomised trials can introduce bias and reduce study power affecting the generalisability, validity, and reliability of results [1]. Many strategies are used by trialists to reduce attrition, including motivating and engaging participants and sites to optimise data return or compliance to follow-up procedures [2].

## Objective

To quantify the effect of strategies to reduce attrition from randomised trials in any healthcare setting.

## Methods

Included studies were randomised evaluations of strategies to reduce attrition embedded within randomised trials from all disease areas and settings. The following sources were searched for eligible studies [3]: MEDLINE (1950 to present), EMBASE (1980 to present), PsycINFO (1806 to present), DARE (most recent issue), CENTRAL (most recent issue), CINAHL (1981 to present), C2-SPECTR (most recent date), and ERIC (1966- present), Cochrane Methodology Register, Current Controlled Trials *meta*Register, WHO trials platform, Society for Clinical Trials (SCT) conference proceedings (1980-2010), and publication reference lists. A survey of all UK clinical trials units (CTU) was also conducted to identify studies.

Two authors reviewed potentially eligible titles and abstracts. Data extracted were checked by two authors. Study investigators were contacted for missing data. Risk of bias was assessed using the Cochrane risk of bias tool. Data were entered into RevMan5 and pooled using the fixed effect model. Heterogeneity was explored to determine whether some types of strategies to reduce attrition were more effective than others. The analyses focused on the primary endpoint of attrition.

## Results

From 19,281 abstracts 31 unique RCTs were identified from the following sources: MEDLINE, CENTRAL, CINAHL n=9; SCT abstracts 1980-2010 n=4; reference lists of relevant reviews n=7; and of included trials n=8 (7 duplicates); word of mouth n=4; and CTUs survey n=6. Six types of strategies to reduce attrition were identified: a) communication i.e. email, letters signed by different study personnel, type of post, and delivery method; b) questionnaire length i.e. short versus long; c) incentives i.e. monetary incentives, offers of monetary incentives or vouchers, and gifts; d) case management i.e. trial assistants assigned to manage participant follow-up; e) behavioural e.g. workshops giving participants information about goal setting; and f) methodological interventions e.g. blinded versus open trials. Final results of the review will be presented**.**

## References

[B1] Fewtrell MS Kennedy K Singhal A Martin RM Ness A Hadders-Algra M How much loss to follow-up is acceptable in long-term randomised trials and prospective studies? Arch Dis Child 2008 93 458 461 10.1136/adc.2007.127316 18495909

[B2] Valerie Brueton C Greta Rait Jayne Tierney Sally Stenning Sarah Meredith Janet Darbyshire Strategies to reduce attrition in randomised trials: A systematic review Clinical Trials 2010 7 418 504

[B3] Brueton VC Rait G Tierney J Meredith S Darbyshire J Harding S Strategies to reduce attrition in randomised trials Cochrane Database of Systematic Reviews Art 2011 No :MR000032 DOI: 10 1002/14651858 MR000032

